# Prognostic implications and interaction of L1 methylation and p53 expression statuses in advanced gastric cancer

**DOI:** 10.1186/s13148-019-0661-x

**Published:** 2019-05-14

**Authors:** Yun-Joo Shin, Younghoon Kim, Xianyu Wen, Nam-Yun Cho, Sun Lee, Woo Ho Kim, Gyeong Hoon Kang

**Affiliations:** 1 Laboratory of Epigenetics, Cancer Research Institute, Seoul National University College of Medicine, Seoul, South Korea; 2 Department of Cancer Biology, Cancer Research Institute, Seoul National University College of Medicine, Seoul, South Korea; 3 Department of Pathology, Seoul National University College of Medicine, 103 Daehak-ro, Chongo-gu, Seoul, 03080 South Korea; 4 Department of Pathology, College of Medicine, Kyung Hee University, Seoul, South Korea

**Keywords:** LINE-1, Methylation, *TP53*, Prognosis, Gastric cancer

## Abstract

**Background:**

*TP53* is frequently mutated across various tissue types of cancers. In normal cells, long interspersed nuclear element-1 (LINE-1, L1) is mostly repressed by DNA methylation in its 5′ untranslated region but is activated by DNA demethylation process during tumorigenesis. p53 is indispensable for maintaining genomic stability and plays its role in controlling genomic stability by repressing retrotransposon activity. However, it is unclear whether p53 regulates expression or methylation of L1 differently depending on the mutational status of *TP53*. Four hundred ninety cases of advanced gastric cancer (AGC) were analyzed for their statuses in p53 expression and L1 methylation using immunohistochemistry and pyrosequencing, respectively. Whether L1 methylation and expression statuses were differently affected by types of *TP*53 mutants was analyzed in gastric cancer cell line.

**Results:**

By p53 immunohistochemistry, tumors were classified into 4 groups according to the intensity and extent of stained tumor nuclei. L1 methylation level was significantly higher in p53 expression group 1 than in the other groups in which L1 methylation level was similar (*P* <  0.001). Although L1 methylation and p53 expression statuses were associated with patient survival, multivariate analysis revealed that L1 methylation was an independent prognostic parameter. In in vitro analysis of AGS cells with the introduction of wild type or mutant types of *TP53*, L1 methylation level and activity were different depending on types of *TP53* mutation.

**Conclusions:**

Findings suggest that L1 methylation level is affected by *TP53* mutation status; although, L1 methylation status was an independent prognostic parameter in patients with AGC. Further study is required to elucidate the mechanism of how wild type or mutant p53 affects L1 activity and methylation status of L1 CpG island.

**Electronic supplementary material:**

The online version of this article (10.1186/s13148-019-0661-x) contains supplementary material, which is available to authorized users.

## Background

Gastric cancer is the fifth most common malignant tumor and the third leading cause of cancer-related death worldwide. Despite advances in technology, the diagnosis and treatment of gastric cancer still remains a challenge [1]. The current TNM staging serves well for the selection of treatment and assessment of prognosis. However, survival time varies in patients with advanced gastric cancer (AGC) within the same stage, which indicates that the current TNM staging system is not sufficient for the prognosis estimation. Development of prognostic biomarkers might ameliorate the prognostication power of the current staging system.

Long interspersed nuclear element-1 (LINE-1, L1) is a retrotransposon which is repeated a half million times in an interspersed manner and comprises about 17% of the human genome. Promoter CpG island hypermethylation is an important mechanism for the suppression of gene expression and retrotransposon activity. Cancer cells tend to undergo diffuse hypomethylation which leads to the increased activity of L1 retrotransposon [2, 3]. Hypomethylation of L1 may contribute to genomic instability and tumorigenesis [4]. DNA hypomethylation in the 5′ untranslated region of L1 repeats has been demonstrated to be closely associated with worse recurrence-free survival and overall survival in patients with gastric cancer [5].

Somatic mutation of the *TP53* gene is one of the most frequent alterations in human cancer [6]. Overall, 50% of human cancers contains *TP53* mutation, and negative regulators of p53, MDM2 and MDM4, are frequently increased in remnant cases [7]. In most cases of *TP53* mutation, a single amino acid is substituted in the DNA binding domain, which leads to loss of function despite the protein length being intact [8, 9]. “Loss of function” mutation in *TP53* means loss of function in the regulation of cell cycle checkpoint and induction of apoptosis by its protein p53 [10]. In spite of the fact that *TP53* is largely accepted as a tumor suppressor gene, oncogenic effect of mutant p53 proteins, including deregulated metabolic pathway, increased tumor invasion, and enhanced chemotherapy resistance, has also been reported, indicating a gain of function role for mutant p53 [10–13]. One of the mechanisms involving gain of function in *TP53* mutation includes upregulation of epigenetic genes, especially genes that serve as histone methyltransferases and acetyltransferases, via binding to the transcription factor. A recent study by Zhu et al. has demonstrated that MLL1, MLL2, and MOZ were upregulated in human tumor samples with *TP53* gain of function mutations, but not when *TP53* was wild type or null status [9]. However, the effect of p53 in other epigenetic regulators, including the promoter methylation status of L1, is still elusive.

To date, it is well established that L1 is hypomethylated in many tissue types of cancer and causes genomic instability. Also, p53 acts as a guardian against transposopathy, which maintains genomic stability of the cell by restraining transposable element such as L1 [14]. However, the correlation between L1 methylation and p53 expression statuses is largely unknown in human gastric cancer. Also, it is unclear whether the difference in mutational status of *TP53* affects L1 expression or not. In the present study, we investigated the correlation between p53 expression and L1 methylation statuses to determine whether expression status or mutational status of *TP53* influences expression and methylation status of L1 in gastric cancer tissues or cell lines.

## Results

### L1 methylation level and p53 expression statuses in AGC

**Fig. 1:**
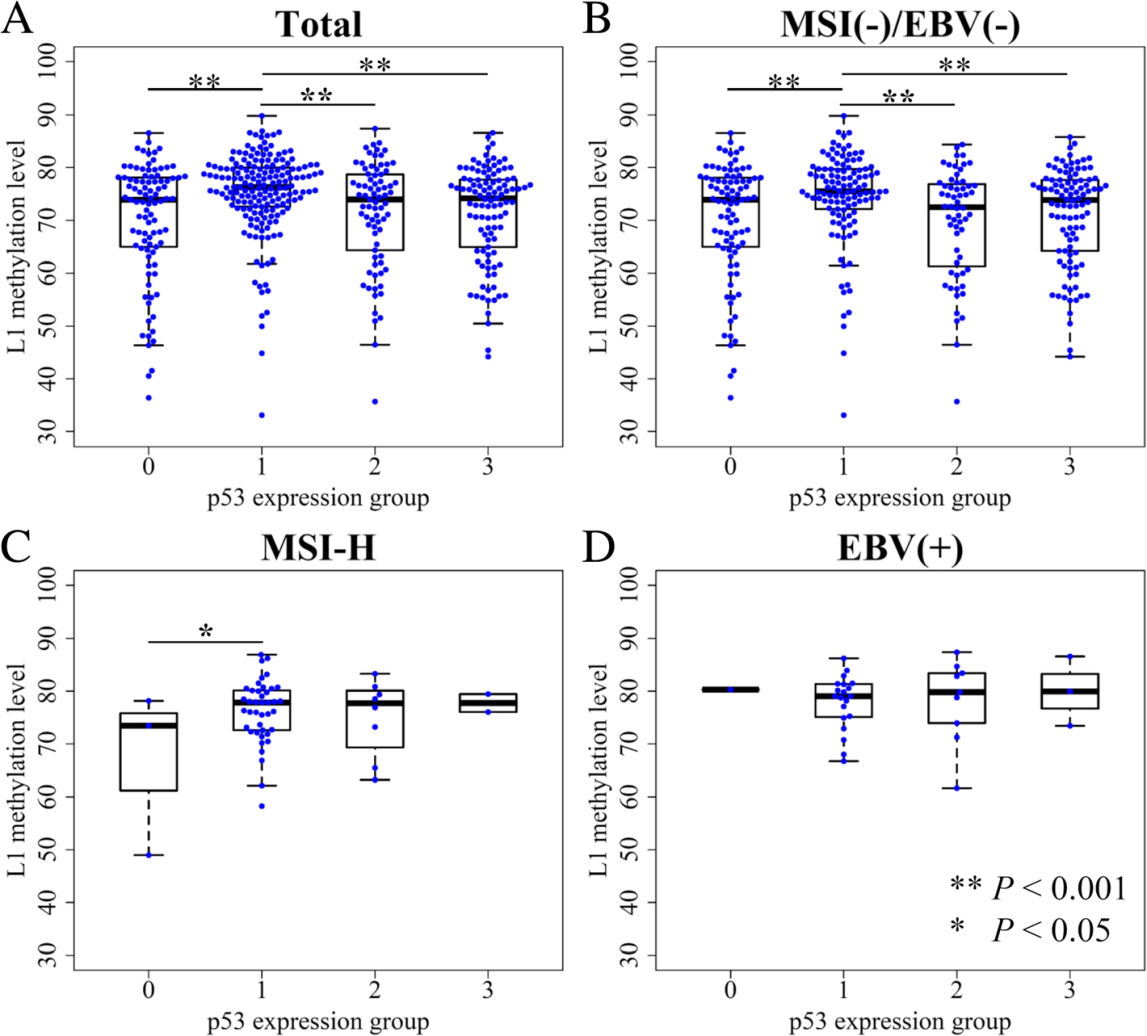
Correlation between p53 expression and L1 methylation level measured by pyrosequencing. **a** All molecular subtype AGC included. **b** MSI-negative, EBV-negative AGC samples, **c** MSI-High AGC samples, **d** EBV-positive AGC samples

p53 expression was categorized into four groups according to the overall intensity of nuclear staining of tumor cells and the extent of stained cells. When we compared L1 methylation levels among four p53 expression groups, significant differences were noted in L1 methylation levels among four subgroups (*P* <  0.001, ANOVA) (Figure 1a). p53 expression group 1 showed a significantly higher level of L1 methylation than that of the other three groups which exhibited similar levels of L1 methylation. Microsatellite instability (MSI)-negative, Epstein–Barr virus (EBV)-negative AGCs also had similar results, as group 1 showed the highest L1 methylation level (Fig. 1b). In MSI-high (MSI-H) and EBV-positive AGC populations, however, a relatively small number of samples for each group undermined the correlation between p53 expression status and L1 methylation level (Fig. 1c & d).

### Correlation between L1 methylation level, p53 expression status, and clinicopathological characteristics

L1 methylation level was significantly associated with various clinicopathological features as we have previously reported (Additional file 1: Figure S1) [5, 15]. Significantly correlated variables include age, sex, lymphatic invasion, venous invasion, Lauren’s classification, pN stage, tumor differentiation, molecular phenotypes, and MSI-H and EBV-positive AGCs. L1 methylation level was 77.3, 73.9, and 71.0% in EBV-positive, MSI-H, and EBV-negative/MSI-negative AGCs, respectively. A significant difference was noted in L1 methylation levels between MSI-H and MSI-negative/EBV-negative AGCs (*P* <  0.001, Student *t* test) and between EBV-positive and MSI-H AGCs (*P* <  0.001, Student *t* test). Correlation between p53 expression and clinicopathological characteristics was measured with two different classification schemes (Table 1). When associations between p53 expression subgroups and clinicopathological features were analyzed, significant associations were found in lymphatic invasion, venous invasion, and MSI and EBV status (*P* < 0.05). Since p53 expression group 1 was contrasted with the other expression groups in terms of L1 methylation level, we compared clinicopathological features between group 1 and the other three groups (group 0, 2, and 3). Group 1 was associated with poor tumor differentiation (*P* < 0.05) but had less lymph node metastasis compared with the other three groups (*P* = 0.008). Moreover, local invasions including lymphatic invasion and venous invasion were much less frequent in group 1 (*P* < 0.001 and *P* = 0.034, respectively). MSI-H and EBV-positive subtypes were more frequently observed in group 1 than in all other groups combined (*P* < 0.001 and *P* = 0.005, respectively).

**Table 1:** p53 expression status and its association with clinicopathological features

	p53 expression group				*P* value	*P* value (1 vs 0, 2, and 3)
	0	1	2	3		
Sex					0.089	0.074
Male	70 (14.3)	117 (23.9)	66 (13.5)	79 (16.1)		
Female	33 (6.7)	69 (14.1)	18 (3.7)	38 (7.8)		
Age					0.927	0.809
> 61	46 (9.4)	91 (18.6)	40 (8.2)	58 (11.8)		
≤ 61	57 (11.6)	95 (19.4)	44 (9.0)	59 (12.0)		
Tumor differentiation					0.174	0.008
Well	2 (0.4)	5 (1.0)	0 (0)	2 (0.4)		
Moderate	27 (5.5)	38 (7.8)	27 (5.5)	32 (6.5)		
Poor	18 (3.7)	72 (14.7)	30 (6.1)	31 (6.3)		
Poorly cohesive	35 (7.1)	53 (10.8)	15 (3.1)	39 (8.0)		
Others	21 (4.3)	18 (3.7)	12 (2.4)	13 (2.7)		
Lauren classification					0.168	0.052
Intestinal	48 (9.8)	58 (5.9)	32 (6.5)	46 (9.4)		
Diffuse	44 (9.0)	99 (11.8)	36 (7.3)	56 (11.4)		
Mixed	11 (2.2)	29 (5.9)	16 (3.3)	15 (3.1)		
pT					0.704	0.390
pT2	26 (5.3)	44 (9)	18 (3.7)	26 (5.3)		
pT3	38 (7.8)	73 (14.9)	31 (6.3)	39 (8.0)		
pT4a	33 (6.7)	63 (12.9)	29 (5.9)	49 (10.0)		
pT4b	6 (1.2)	6 (1.2)	6 (1.2)	3 (0.6)		
pN					0.056	0.008
pN0	29 (5.9)	72 (14.7)	22 (4.5)	25 (5.1)		
pN1	12 (2.4)	34 (6.9)	18 (3.7)	27 (5.5)		
pN2	22 (4.5)	30 (6.1)	19 (3.9)	21 (4.3)		
pN3a	26 (5.3)	27 (5.5)	18 (3.7)	27 (5.5)		
pN3b	14 (2.9)	23 (4.7)	7 (1.4)	17 (3.5)		
pTNM					0.407	0.090
IB	16 (3.3)	30 (6.1)	7 (1.4)	10 (2.0)		
IIA	13 (2.7)	39 (8.0)	12 (2.4)	16 (3.3)		
IIB	11 (2.2)	26 (5.3)	21 (4.3)	24 (4.9)		
IIIA	13 (2.7)	27 (5.5)	7 (1.4)	12 (2.4)		
IIIB	18 (3.7)	21 (4.3)	14 (2.9)	21 (4.3)		
IIIC	18 (3.7)	23 (4.7)	15 (3.1)	22 (4.5)		
IV	14 (2.9)	20 (4.1)	8 (1.6)	12 (2.4)		
Lymphatic invasion					< 0.001	< 0.001
Absent	29 (5.9)	93 (19.0)	27 (5.5)	31 (6.3)		
Present	74 (15.1)	93 (19.0)	57 (11.6)	86 (17.6)		
Venous invasion					0.028	0.034
Absent	66 (13.5)	149 (30.4)	61 (12.5)	89 (18.2)		
Present	37 (7.6)	37 (7.6)	23 (4.7)	28 (5.7)		
Perineural invasion					0.051	0.305
Absent	56 (11.4)	78 (15.9)	42 (8.6)	44 (9.0)		
Present	47 (9.6)	108 (2.0)	42 (8.6)	73 (14.9)		
MSI					< 0.001	< 0.001
Stable	86 (17.6)	128 (2.4)	71 (14.5)	98 (20.0)		
Low	14 (2.9)	13 (2.7)	5 (1.0)	16 (3.3)		
High	3 (0.6)	45 (9.2)	8 (1.6)	3 (0.6)		
EBV					< 0.001	< 0.001
Absent	102 (20.8)	164 (33.5)	74 (15.1)	114 (23.3)		
Present	1 (0.2)	22 (4.5)	10 (2.0)	3 (0.6)		

Percentages in parenthesis

*MSI* microsatellite instability, *EBV* Epstein–Barr virus

### Survival analysis

**Fig. 2:**
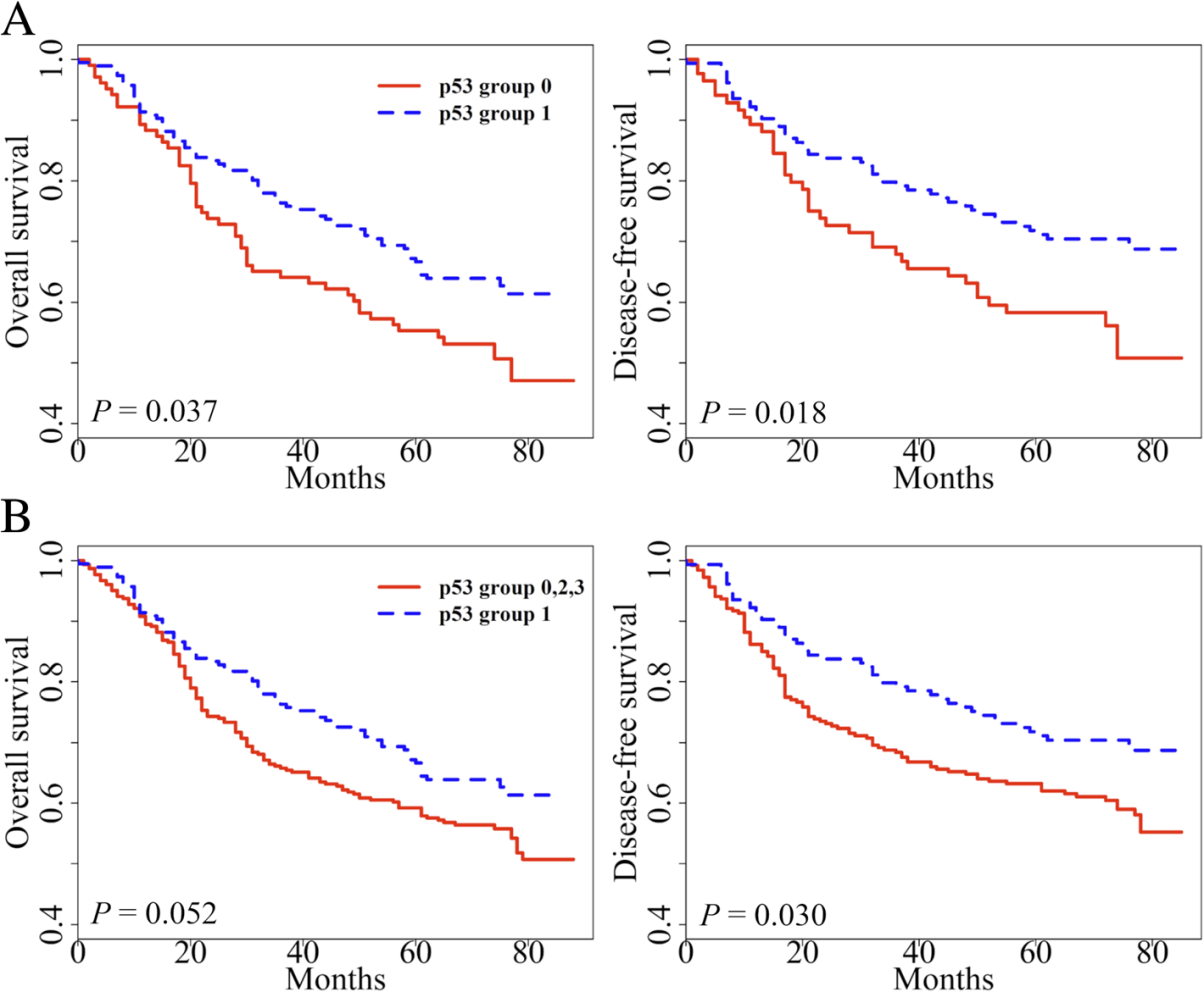
Kaplan–Meier curve with a log-rank test for p53 expression status. **a** p53 expression group 1 versus group 0 in overall survival (OS) and disease-free survival (DFS), **b** All AGC samples were divided into two categories (p53 expression group 1 versus group 0, 2, and 3) for survival analysis of overall survival (OS) and disease-free survival (DFS)

**Fig. 3:**
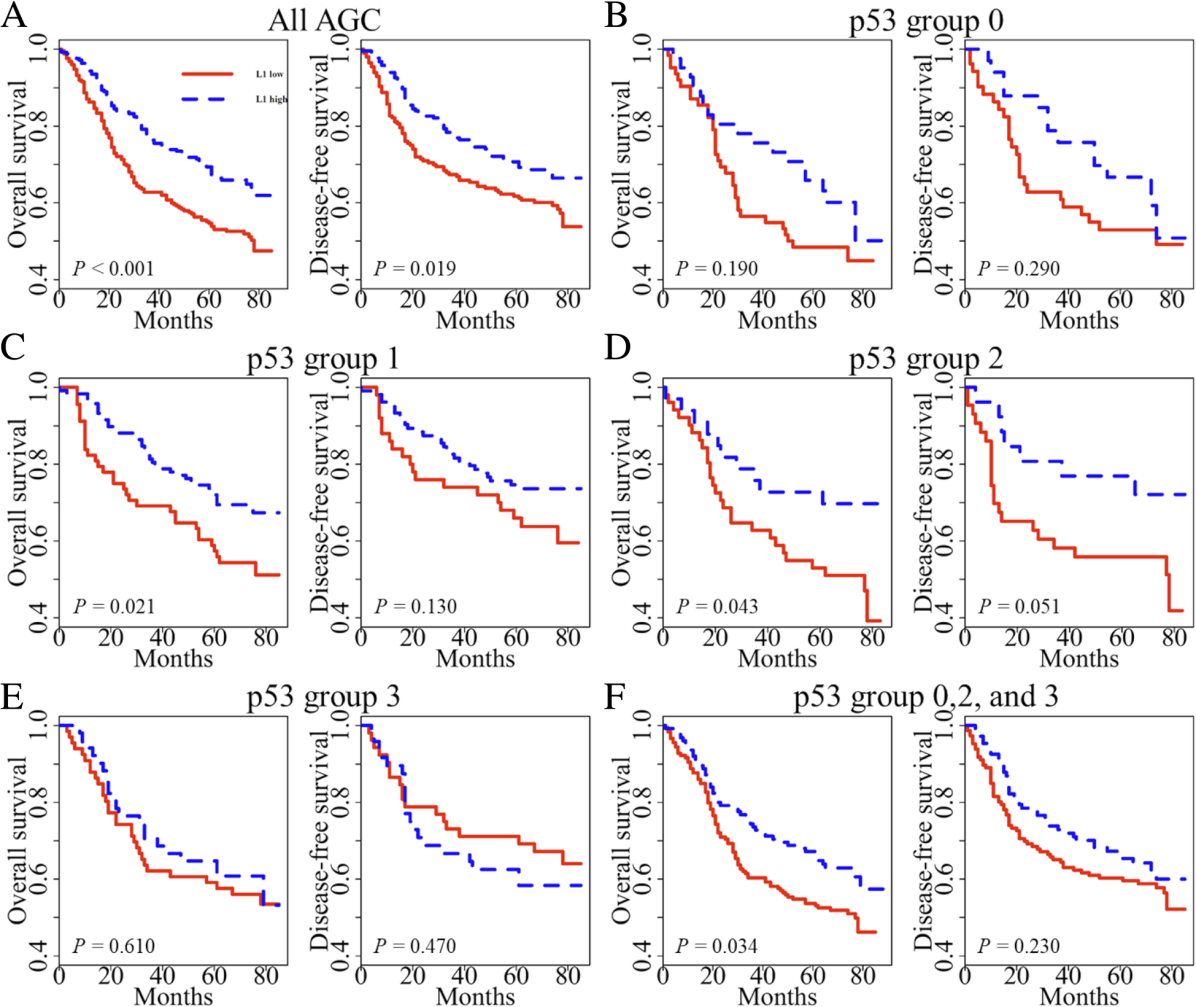
Kaplan–Meier survival curves were compared between two groups using a log-rank test to determine difference of patient survival according to L1 methylation status in each p53 expression group. **a** All AGC samples were included. **b** AGC samples with negative p53 expression (intensity group 0). **c** AGC samples with p53 expression group 1. **d** AGC samples with p53 expression group 2. **e** AGC samples with p53 expression group 3. **f** All AGC samples except p53 expression group 1 (group 0, 2, and 3)

In previous studies [5, 15], we have found that tumoral L1 hypomethylation is a biomarker associated with poor prognosis. However, the effect of p53 has not been considered as a covariate in these attempts. Therefore, we further explored the prognostic implication of L1 methylation with the addition of p53 expression status. L1 methylation status was classified into low and high as previously described [15]. In univariate survival analysis, p53 group 1 showed prolonged overall survival (OS) and disease-free survival (DFS) when compared with the respective one of p53 group 0 (*P* = 0.037 and *P* = 0.018, respectively; Fig. 2a). When p53 group 1 was compared with all other groups combined, p53 group 1 exhibited marginally better OS than all other p53 groups (*P* = 0.052, Fig. 2b). p53 group 1 also had a favorable prognosis for DFS compared with all other p53 groups (*P* = 0.030). Multivariate analysis reveals that L1 methylation level, lymphatic invasion, and pTNM stage were independent prognostic factors in OS and DFS. On the other hand, p53 expression status was not a prognostic factor when adjusted for pTNM stage, lymphatic invasion, venous invasion, perineural invasion, and L1 methylation level (Table 2). Also, we analyzed patient survival according to L1 methylation status in each p53 subset (Fig. 3). These findings confirmed that L1 methylation level was a superior prognostic marker in AGC. However, we clearly identified the potential interaction between p53 expression and L1 methylation (Fig. 1). From these findings, we decided to investigate the interaction of p53 expression and L1 methylation in vitro.

**Table 2:** Multivariate survival analysis

Variables	Hazard ratio	95% CI	*P* value
Overall survival			
pTNM	1.955	1.429–2.673	< 0.001
Lymphatic invasion	1.719	1.126–2.624	0.012
Venous invasion	1.349	0.936–1.946	0.109
Perineural invasion	1.228	0.862–1.750	0.255
p53 expression status	0.877	0.599–1.285	0.501
L1 methylation	0.566	0.381–0.841	0.005
Disease-free survival			
pTNM	1.963	1.435–2.684	< 0.001
Lymphatic invasion	1.758	1.149–2.689	0.009
Venous invasion	1.329	0.918–1.924	0.132
Perineural invasion	1.145	0.806–1.628	0.449
p53 expression status	0.859	0.587–1.255	0.432
L1 methylation	0.979	0.962–0.996	0.015

### In vitro study demonstrating variation of L1 methylation level according to p53 mutation status

**Fig. 4:**
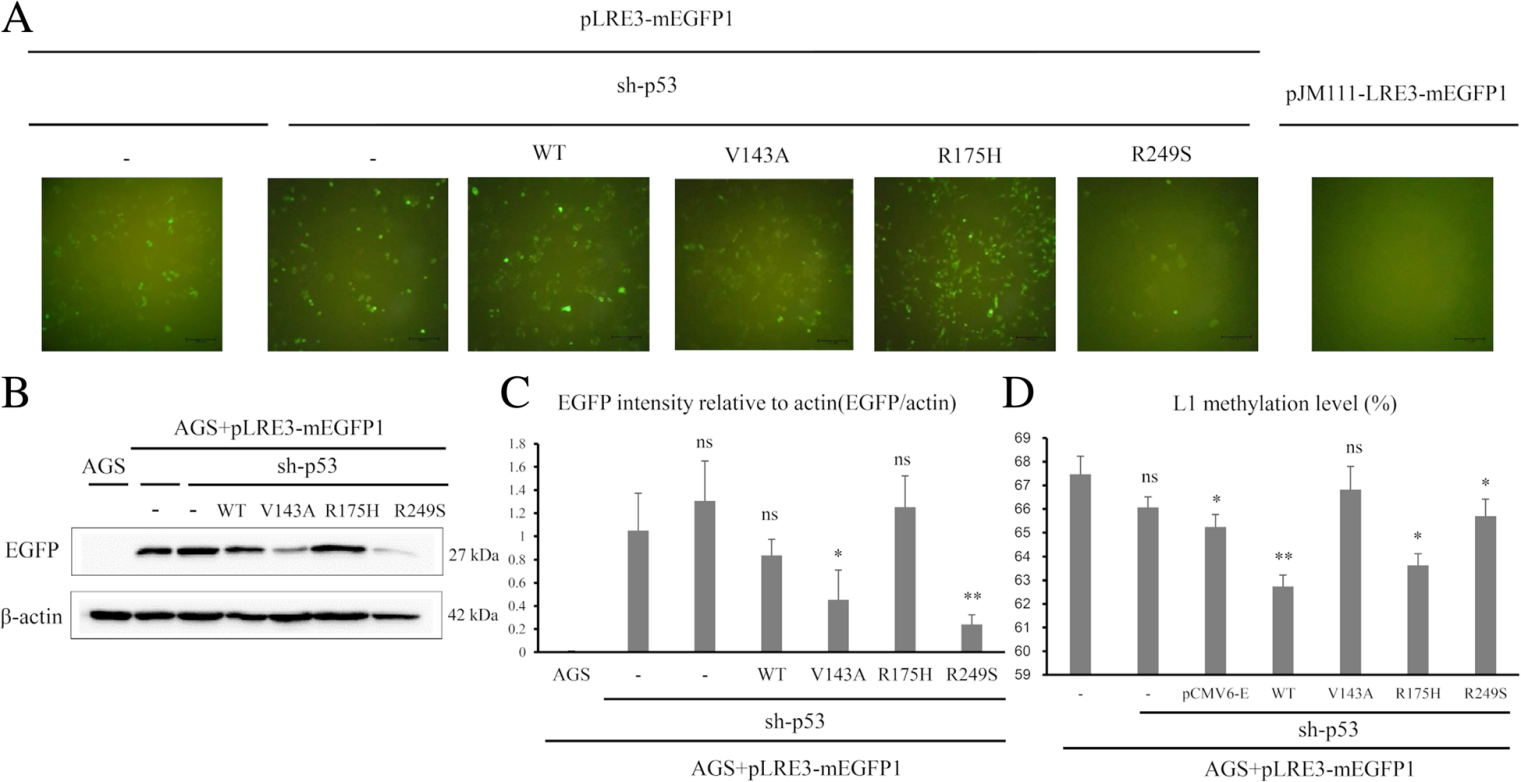
L1 expression level and methylation level in *TP53*-transfected AGS gastric cancer cell line. **a** Green fluorescence protein observed by fluorescence microscope from *TP53*-transfected AGS (scale bar 100 μm). **b** EGFP protein expression levels from *TP53*-transfected AGS. **c** Quantification of EGFP intensity by western blot from *TP53*-transfected AGS. **d** Mean methylation levels of the four L1 CpG sites for AGS cell line expressing wild type and mutant type *TP53* (V143A, R175H, and R249S). (ns *P* > 0.05, *P < 0.05, **P < 0.01)

Gastric cancer cell line AGS was found to be wild type *TP53* alleles (http://www.cbioportal.org). AGS cells were transfected with pLRE3-mEGFP1 (wild type L1 plasmid) and then knocked down with *TP53* shRNA to generate cell status comparable to null mutation of *TP53* in terms of p53 expression status. *TP53*-knocked down AGS cells were stably transfected with wild type *TP53* or mutant type *TP53* (V143A, R175H, and R249S) (Additional file 2: Figure S2). We assessed L1 activity by evaluation of EGFP expression since it is difficult to detect ORF proteins of L1 due to its unconventional translation mechanism [16, 17]. Therefore, EGFP expression was considered as a surrogate for L1 expression (Fig. 4). More EGFP-positive cells were observed in AGS cells transfected with mutant *TP53* R175H cells than in AGS cells transfected with mutant *TP53* V143A or R249S, which was confirmed by western blot of EGFP protein.

## Discussion

In our previous studies, L1 methylation level was found to be a reliable independent prognostic biomarker in which a low methylation status was associated with worse clinical outcome of AGC patients (17, 18). In our current investigation, L1 methylation level was different among p53 expression groups which were also associated with OS and DFS. Despite a strong correlation between L1 methylation level and p53 expression status, tumoral L1 hypomethylation was found to be an independent prognostic parameter in a multivariate analysis incorporating p53 expression status as a covariate. However, p53 expression status no longer maintained its prognostic implication in multivariate analysis. Although tumoral L1 hypomethylation was a prognostic parameter, L1 methylation status did not show any prognostic difference of survival in patients with AGC of p53 expression group 3. This finding indicates that application of L1 methylation status as a prognostic biomarker needs to be accompanied with p53 immunohistochemistry.

In the present study, we did not perform sequencing analysis of *TP53* mutation and instead used p53 expression status (determined by immunohistochemistry) as surrogates for *TP53* mutation status. In a previous study in which we analyzed colorectal cancer tissue for their *TP53* mutation and expression statuses using next-generation sequencing and immunohistochemistry, respectively, and correlated them, we found that p53 expression groups differed significantly in the frequency and type of *TP53* mutations; nonsynonymous single-nucleotide variant rate was 80%, 12%, and 2% in p53 group 3, 2, and 1, respectively, whereas p53 group 0 was featured with stop-gain mutations (38%) and indels (15%) [18]. Through the grouping of p53 immunohistochemistry, it was possible to predict what percentage of variants are likely to be and what kind of mutation will be present, but it is not correct to predict that group 1 has no mutation or group 0 does not have wild type. Other researchers also have reported that a strong correlation exists between gene mutation and p53 expression group [19, 20]. Therefore, we deduced that the immunohistochemical status of p53 is a representative of its mutation status. However, limitation of the utilization as p53 expression status as surrogates for TP53 mutation status is as follows: with immunohistochemical finding, it cannot be predicted whether TP53 is wild type or mutated, but the probability of mutation can be predicted based on grouping of p53 expression.

In our study, three expression vectors encoding DNA-binding domain p53 mutants (V143A, R175H, and R249S) were transfected to explore whether p53 mutants differentially influence L1 activity and methylation. L1-transfected AGS cells showed different L1 activity and methylation level depending on the type of p53 mutants. Mutant p53 R175H and R249S are among the p53 hotspot mutants and these arginine residues (Arg-175 and Arg-249) play a role in maintaining the structural integrity of the DNA binding surface [21]. Although mutant p53 V143A is not one of the p53 hotspot mutants, valine residue (Val-143) is located in the hydrophobic core of the beta-sandwich region and V143A mutant leads to destabilization of the three-dimensional structure and temperature sensitivity of p53 binding to many response elements [21]. Furthermore, mutant p53 R175H and R249S are known to lose their DNA binding affinity to *GADD45* DNA, whereas mutant p53 V143A retains its binding affinity to *GADD45* DNA [22]. In the present study, mutant p53 R175H shows higher L1 activity and lower L1 methylation level compared with other mutant p53 V143A and R249S.

In the present study, we found that tumoral L1 hypomethylation did not have prognostic implications in patients with gastric cancer of p53 group 3. This finding suggests that endogenous factors of tumor cells might have an influence on prognostic implications of tumoral L1 hypomethylation and that p53 immunohistochemistry is required for the application of L1 methylation status as a prognostic parameter in AGC. Besides gastric cancer, several tissue types of human cancer have shown the association between tumoral L1 hypomethylation and shortened survival time of patients, including esophageal squamous cell carcinoma, lung cancer, colorectal cancer, and intrahepatic cholangiocarcinoma [23–27]. These tissue types of human cancer are known to have a high frequency of *TP53* mutation. However, limited information is available regarding the interaction of *TP53* in the relationship between tumoral L1 hypomethylation and shortened survival time of tumor patients. Although Morikawa et al.’s study has demonstrated a difference of L1 methylation level between CRCs of p53 group 0 and 1 and CRCs of group 2 and 3, multivariate analysis did not reveal such a relationship [28]. Morikawa et al.’s study did not separate CRCs of p53 group 0 and group 1 and instead grouped together into a p53-negative group, which might underestimate the relationship between L1 methylation level and p53 expression group. Nevertheless, L1 methylation and p53 expression statuses were found to be independently associated with colorectal cancer outcome. However, in the present study, p53 expression status was not an independent prognostic factor in patients with AGC, while L1 methylation status was an independent prognostic parameter.

In a previous study in which Kawakami et al. analyzed microsatellite-stable and CpG island methylator phenotype-negative colorectal cancers (*n* = 131) for their L1 methylation status and investigated the influence of adjuvant fluopyrimidines on the prognostic value of L1 methylation status [29], CRC patients with low L1 methylation status who were treated with adjuvant fluoropyrimidines showed better survival than that of patients with low methylation status, treated with surgery alone. Such a survival benefit from adjuvant fluoropyrimidines was not found for CRC patients with high L1 methylation status. Thus, a question might be raised upon whether adjuvant chemotherapy might exert an influence on the prognostic value of L1 methylation status in AGC patients of the present study. When OS and DFS were analyzed for their associations with L1 methylation status in four p53 expression groups, the tendency of low L1 methylation status toward worse survival was observed in groups 0, 1, and 2 regardless of whether the patients received adjuvant chemotherapy or not (Additional file 3: Figure S3). These results suggest that adjuvant chemotherapy might not influence the prognostic value of L1 methylation status in patients with AGC. For group 3, L1 methylation status was not associated with survival in patients treated with adjuvant chemotherapy or surgery alone.

## Conclusions

Taken together, we have found that *TP53* mutation status affects L1 methylation level and retrotransposon activity in tumor cells and that L1 methylation level was different in AGCs according to p53 expression status. Our findings suggest that application of tumoral L1 hypomethylation as a prognostic parameter to gastric cancer should be accompanied by p53 immunohistochemistry although tumoral L1 hypomethylation was an independent prognostic parameter heralding poor prognosis.

## Methods

### Patient and tissue specimens

Four hundred ninety formalin-fixed paraffin-embedded (FFPE) samples of AGC, defined by gastric cancer with the invasive depth of at least the muscularis propria (pT2–pT4 according to the 7th cancer staging system of the American Joint Committee on Cancer), were collected from the pathological archive of Seoul National University Hospital. All samples were selected from patients who received resection of AGC between January 2007 and December 2008. Clinical data were obtained from the electronic medical record retrospectively. The age of patients ranged from 23 to 86 (mean age of 61). Male to female ratio was 2.10:1. This study was approved by the Institutional Review Board of Seoul National University Hospital, which waived the requirements to obtain informed patient consent.

### Detection of molecular subtypes

All AGC samples were tested for MSI and EBV infection. For MSI status, 5-marker scoring panel (*BAT25, BAT26, D2S123, D5S345, and D17250*) was applied. MSI-H was defined when instability was detected in greater than or equal to 40% of markers. Other cases were categorized as MSI-negative. EBV-positive AGC was detected via in situ hybridization with RNAscope FFPE assay kit (# 300039, ACDbio Inc., Hayward, CA, USA) that target *EBER1*.

### Extraction of genomic DNA from archival tissue samples

FFPE blocks containing each patient sample were cut with 10-μm thickness and attached to glass slides. Slides were soaked into xylene followed by air dry. Tumor areas of each slide were traced from tumor areas marked from its H&E counterpart and microdissected with a razor blade into 50 μL of lysis buffer including 10% proteinase K (P4850, Sigma-Aldrich, St. Louis, MO, USA). Dissected tissues were incubated at 56 °C for at least 24 h. Proteinase K was inactivated by heat block at 95 °C for 30 min.

### Tissue microarray (TMA) and immunohistochemistry

Through microscopic examination, representative areas that contain a considerable amount of tumor were selected and core tissues (2 mm in diameter) were extracted for each AGC FFPE sample and constructed into TMA blocks. For immunohistochemical staining, sections of TMA were immunostained after antigen retrieval. Primary antibody for anti-p53 (clone DO-7, DAKO, Santa Barbara, CA, USA) was stained at a concentration of 1:1000. The proportion of tumor cells with moderate/strong nuclear staining for *TP53* was estimated by light microscopic examination of two tissue cores (2 mm in diameter) for each patient sample. Tumors were defined as p53 group 3 and 2 when > 90% and 90–50% of tumor cells showed moderate/strong nuclear staining, respectively. p53 group 1 denoted samples with moderate/strong nuclear staining in less than 50% of tumor cells or samples with weak nuclear staining. p53 negativity (p53 group 0) was defined as no staining of tumor cells which was contrasted with weak nuclear staining of interstitial lymphocytes (Additional file 4: Figure S4).

### Cell culture and growth media

The human gastric cancer cell line AGS was purchased from Korean Cell Line Bank (KCLB# 21739, Korean Cell Line Bank, Seoul, Korea) and was grown in RPMI-1640 (LM 011–01, Welgene Co., Daegu, Korea) supplemented with 10% heat-inactivated FBS (Fetal bovine serum) (26140–079, Gibco, Grand Island, NY, USA), 100 U/ml penicillin, and 100 μg/ml streptomycin (15070–063, Gibco).

### Plasmid DNA used in transfection assay

Plasmids encoding wild type *TP53* (#16434, Addgene, Watertown, MA, USA) and mutant type *TP53* (V143A, R175H, and R249S) (#16435, #16436, and #16438, Addgene) under the control of the CMV promoter were purchased from Addgene. The pLRE3-mEGFP1 (monomeric enhanced green fluorescent protein) plasmid, encoding a retrotransposition-competent L1, and pJM111-LRE3-mEGFP1, encoding a retrotransposition-defective L1, were generous gifts from Dr. Moran (University of Michigan Medical school). pCMV6-Entry (PS100001, ORIGENE, Rockville, MD, USA) (that includes G418-resistance gene) plasmid was used as a control DNA. Schematic diagram of the pLRE3-mEGFP1 construct and rationale of the L1-retrotransposition assay is depicted in Additional file 5: Figure S5.

### p53 knockdown using shRNA lentiviral plasmid

Plasmids expressing control shRNA (short hairpin RNA) (#H1(shRNA-Ctr)-RB) and shRNA against human *TP53* (#LVP343-RB) were purchased from GenTarget Inc. (San Diego, CA, USA). The plasmid contains RFP (Red Fluorescence Protein)-Bsd (Blasticidin) dual selection marker (15,205, Sigma-Aldrich). AGS cells were transduced with pre-made lentiviral plasmid containing control shRNA and *TP53* shRNA. Three days after transduction, cells were incubated with RPMI-1640 complete medium containing 6 μg/ml blasticidin (RPMI-Bsd). For generating cells stably expressing control shRNA and *TP53* shRNA, single cells expressing RFP were sorted by BD FACS (Fluorescence-activated cell sorter) Aria II into individual wells of 96-well plates. Several single colonies were screened by light microscope, selected, and transferred to individual wells in 48-well dishes and expanded in RPMI-Bsd media. Stable knockdown of *TP53* expression was confirmed by western blot.

### Generation of stable cell line (transfection and selection of cells)

AGS cells were transfected with the pLRE3-mEGFP1 and pJM111-LRE3-mEGFP1 construct by electroporation (Nepa Gene Co., Ichikawa city, Japan) and selected for puromycin-resistant cells by growth in RPMI-1640 complete medium containing 0.3 μg/ml puromycin (RPMI-Puro) (P8833, Sigma-Aldrich). After complete antibiotic selection, the cells were trypsinized and plated at low density in 6-well plates. EGFP-expressing clones were observed by fluorescence microscopy. Several fluorescent colonies were selected, transferred to individual wells in 6-well dishes, and expanded in RPMI-Puro. AGS cells stably expressing L1 were transduced with a lentiviral plasmid expressing *TP53* shRNA and single cells were sorted by BD FACS (Fluorescence-activated cell sorter) Aria II. Wild type and three types of mutant *TP53* were then transfected into AGS stably expressing L1 and p53 shRNA. G418 (A1720, Sigma-Aldrich) selection and confirmation of transfection were done as described above. The colonies were screened for their presence by western blot analysis.

### Western blot analysis

Cells were lysed with RIPA buffer supplemented with protease inhibitor cocktail (11,836,153,001, Complete Mini, Roche, Mannheim, Germany) and phosphatase inhibitor cocktail (04906845001, PhosSTOP EASYpack, Roche). Cell debris were then removed by centrifugation at 4 °C. After centrifugation, protein concentrations were measured with Pierce BCA Protein Assay Kit (23,225, Thermo Fisher Scientific, Rockford, IL, USA). For western blotting, 30 μg of protein lysates from each established cell line were separated by 8~10% SDS-polyacrylamide gel electrophoresis (PAGE), transferred onto nitrocellulose membranes (IPVH00010, Millipore, Bedford, MA, USA), blocked at room temperature for 1 h with 5% skim milk, and incubated at 4 °C overnight with the following primary antibodies: p53 (1:1000, sc-126, Santa Cruz Biotechnology, CA, USA), β-actin (1:1000, sc-47,778, Santa Cruz Biotechnology), and EGFP (1:1000, ab184601, Abcam, Cambridge, UK). After three 15-min washes with Tris-buffered saline containing 0.1% Tween 20, the blots were incubated at room temperature for 1 h with horseradish peroxidase-conjugated secondary antibody: goat anti-Mouse IgG(H+L)-HRP (1:4000, #SA001–500, GenDEPOT, Barker, TX, USA). Proteins were detected using chemiluminescent reagent, ECL solution (#W6002, Biosesang, Gyeonggi, Korea).

### DNA preparation and bisulfite conversion

Genomic DNA was extracted from cell lines using the QIAamp DNA mini kit (51,306, Qiagen, Hilden, Germany). DNA samples were then digested in 20-μl reaction volumes with 15 U of HindIII (1060A, Takara Shuzo Co., Kyoto, Japan) for 1 h at 37 °C prior to bisulfite modification. Bisulfite modification was performed using the Zymo EZ DNA methylation Kit (D5002, Zymo Research, Irvine, CA, USA) with 500 ng of digested genomic DNA according to the manufacturer’s protocol.

### MethyLight assay and pyrosequencing methylation assay

After bisulfite modification, Alu-based MethyLight control reaction, a CpG-independent and bisulfite-specific control reaction, was performed to measure input DNA (bisulfite-modified DNA) [30]. We determined the threshold cycle (C(t) value) of this reaction in which the Alu reaction fluorescence was detected. To keep the C(t) value of bisulfite-modified DNA samples in the range of 18 to 20, distilled water was added to dilute bisulfite-modified DNA samples with C(t) values lower than 18. The sodium bisulfite-modified DNA samples were amplified with the same oligonucleotide primers which were designed against a consensus L1 sequence by the Issa group for pyrosequencing [31]. Pyrosequencing methylation assay was performed as described previously [32].

### Statistical analysis

All statistical analysis was performed by R (version 3.5.1). The correlation between categorical variables was performed with chi-square test when all categories were more than 5. If at least one category was equal to or less than 5, Fisher exact test was applied. Correlation between continuous variables and two categories were measured by Student *t* test. ANOVA was performed between variables that included more than two categories. Mann–Whitney U test (Wilcoxon rank-sum test) was applied when the relationship between continuous variables and variables with more than two categories was measured. Univariate survival was determined by Kaplan–Meier curve with a log-rank test. To calculate multivariate survival, Cox regression model was applied. *P* value of < 0.05 was considered as significant.

## Additional files

Additional file 1: **Figure S1.** Forest plot displaying relationships between L1 methylation level and clinicopathological characteristics. (TIF 400 kb)

Additional file 2: **Figure S2.** Evaluation of transfection by western blot analysis. p53 protein expression level in AGS transfected with wild type or mutant types of *TP53* by western blot. (TIF 324 kb)

Additional file 3: **Figure S3.** Comparative analyses of overall survival and disease-free survival in four p53 expression groups of gastric cancers according to L1 methylation status with separation into adjuvant chemotherapy-treated and nontreated groups. (TIF 4730 kb)

Additional file 4: **FigureS4.** Classification of p53 expression status by immunohistochemical staining. (A) p53 negativity (group 0). (B) Moderate/strong nuclear staining in less than 50% of tumor cells or samples with weak nuclear staining (group 1). (C) 90–50% of tumor cells showed moderate/strong nuclear staining (group 2). (D) > 90% of tumor cells showed moderate/strong nuclear staining (group 3). (TIF 4060 kb)

Additional file 5: **Figure S5.** Schematic diagram of the pLRE3-mEGFP1 construct (left) and rationale of the L1-retrotransposition assay (right). The EGFP retrotransposition reporter cassette is cloned into the 3′UTR of L1 in the antisense orientation. The cassette consists of the CMV promoter (pCMV), the TK poly(A) signal (pA) and the EGFP gene interrupted by a sense orientation intron (intron) with the splice donor (SD) and splice acceptor (SA). (TIF 335 kb)

## Abbreviations

AGC: Advanced gastric cancer
DFS: Disease-free survival
EBV: Epstein–Barr virus
FFPE: Formalin-fixed paraffin embedded
L1: Long interspersed nuclear element-1 (LINE-1)
MSI: Microsatellite instability
OS: Overall survival
TMA: Tissue microarray
