# Supplementary figures and images for: Prognostic implications and interaction of L1 methylation and p53 expression statuses in advanced gastric cancer
Source: Clin Epigenetics. 2019 May 14;11:77. doi: 10.1186/s13148-019-0661-x (PMC6518708; doi:10.1186/s13148-019-0661-x)

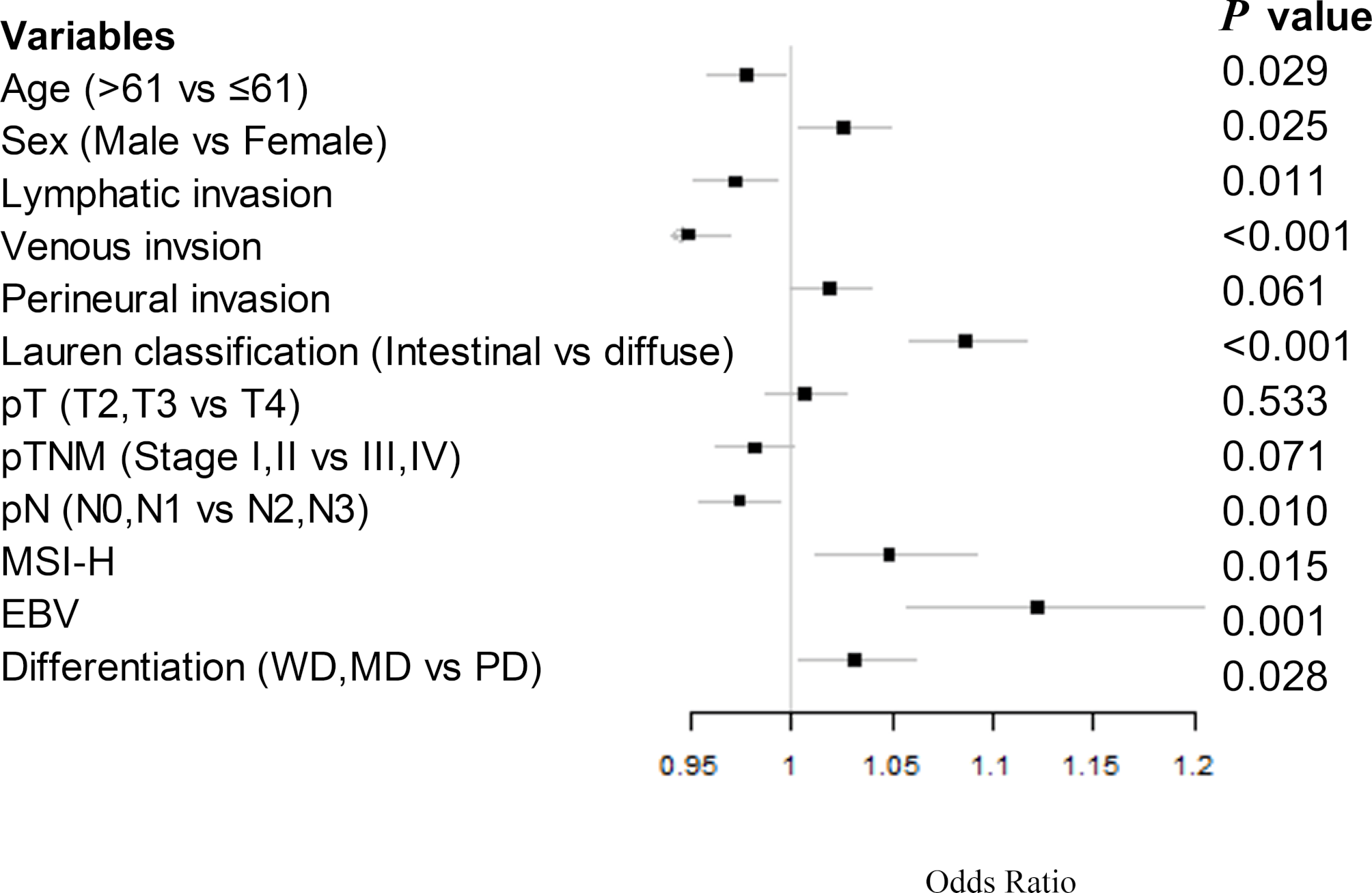

Supplement: Supplementary file 1 — Figure S1. Forest plot displaying relationships between L1 methylation level and clinicopathological characteristics. (TIF 400 kb) [file 13148_2019_661_MOESM1_ESM.tif]

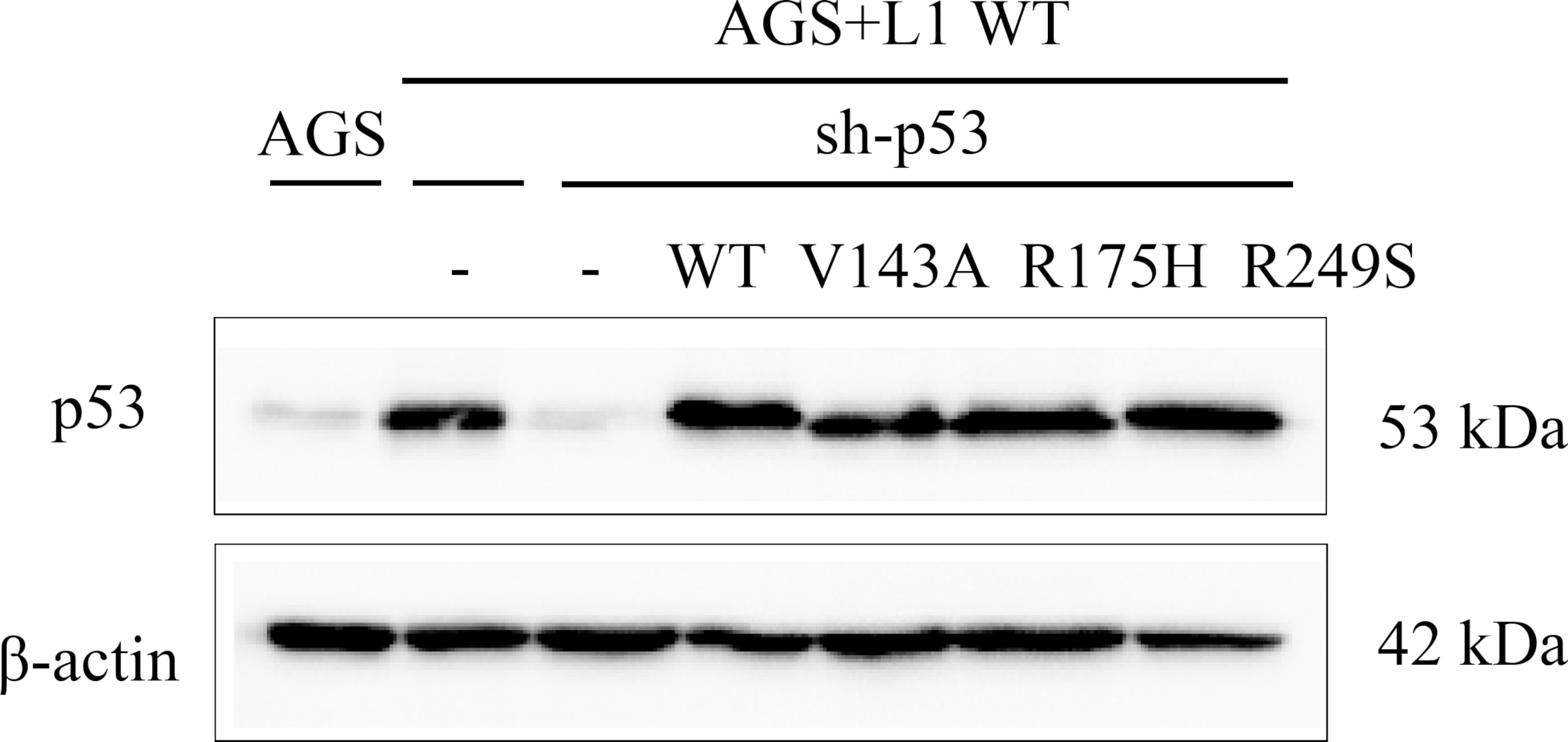

Supplement: Supplementary file 2 — Figure S2. Evaluation of transfection by western blot analysis. p53 protein expression level in AGS transfected with wild type or mutant types of TP53 by western blot. (TIF 324 kb) [file 13148_2019_661_MOESM2_ESM.tif]

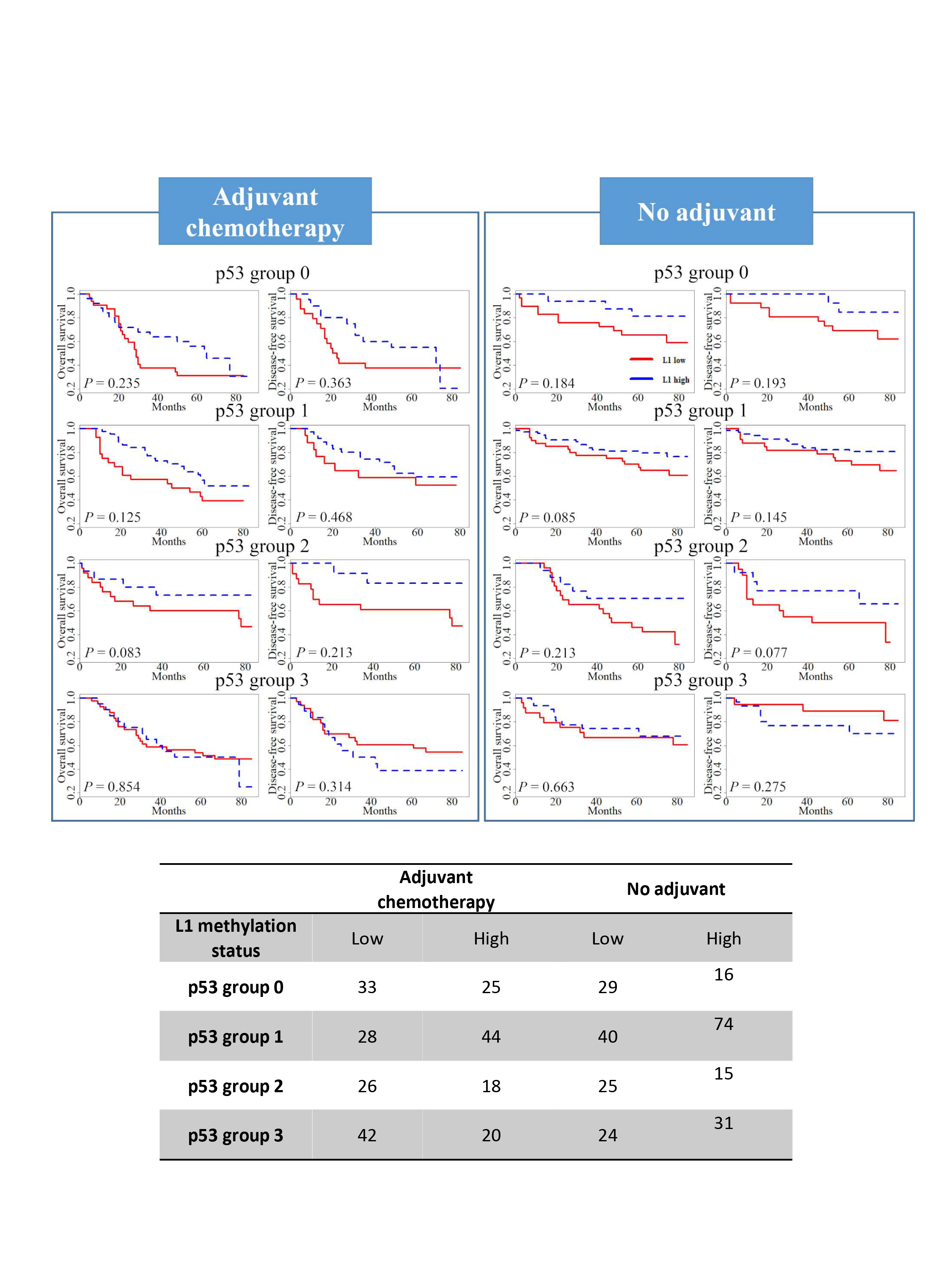

Supplement: Supplementary file 3 — Figure S3. Comparative analyses of overall survival and disease-free survival in four p53 expression groups of gastric cancers according to L1 methylation status with separation into adjuvant chemotherapy-treated and nontreated groups. (TIF 4730 kb) [file 13148_2019_661_MOESM3_ESM.tif]

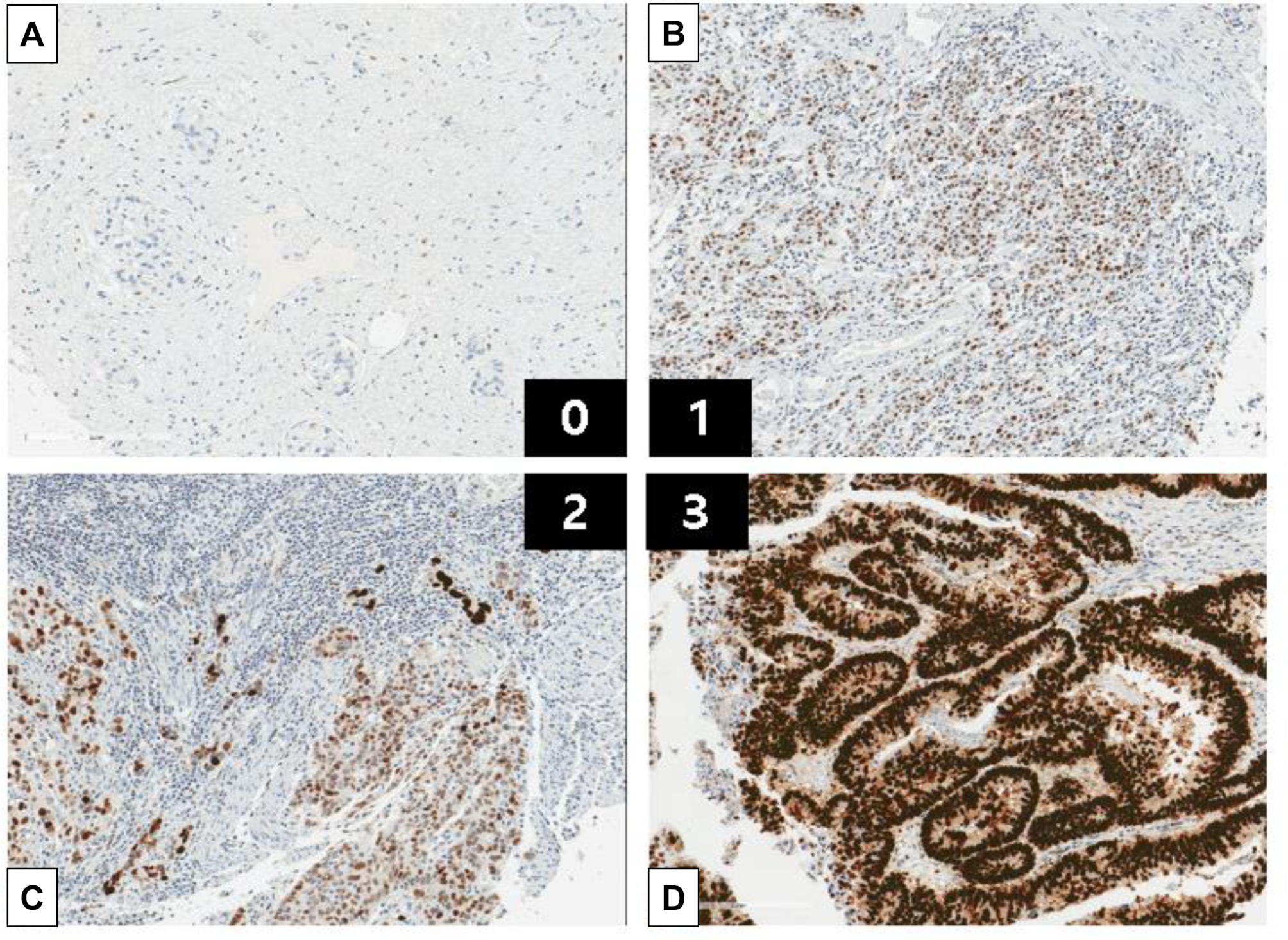

Supplement: Supplementary file 4 — FigureS4. Classification of p53 expression status by immunohistochemical staining. (A) p53 negativity (group 0). (B) Moderate/strong nuclear staining in less than 50% of tumor cells or samples with weak nuclear staining (group 1). (C) 90–50% of tumor cells showed moderate/strong nuclear staining (group 2). (D) > 90% of tumor cells showed moderate/strong nuclear staining (group 3). (TIF 4060 kb) [file 13148_2019_661_MOESM4_ESM.tif]

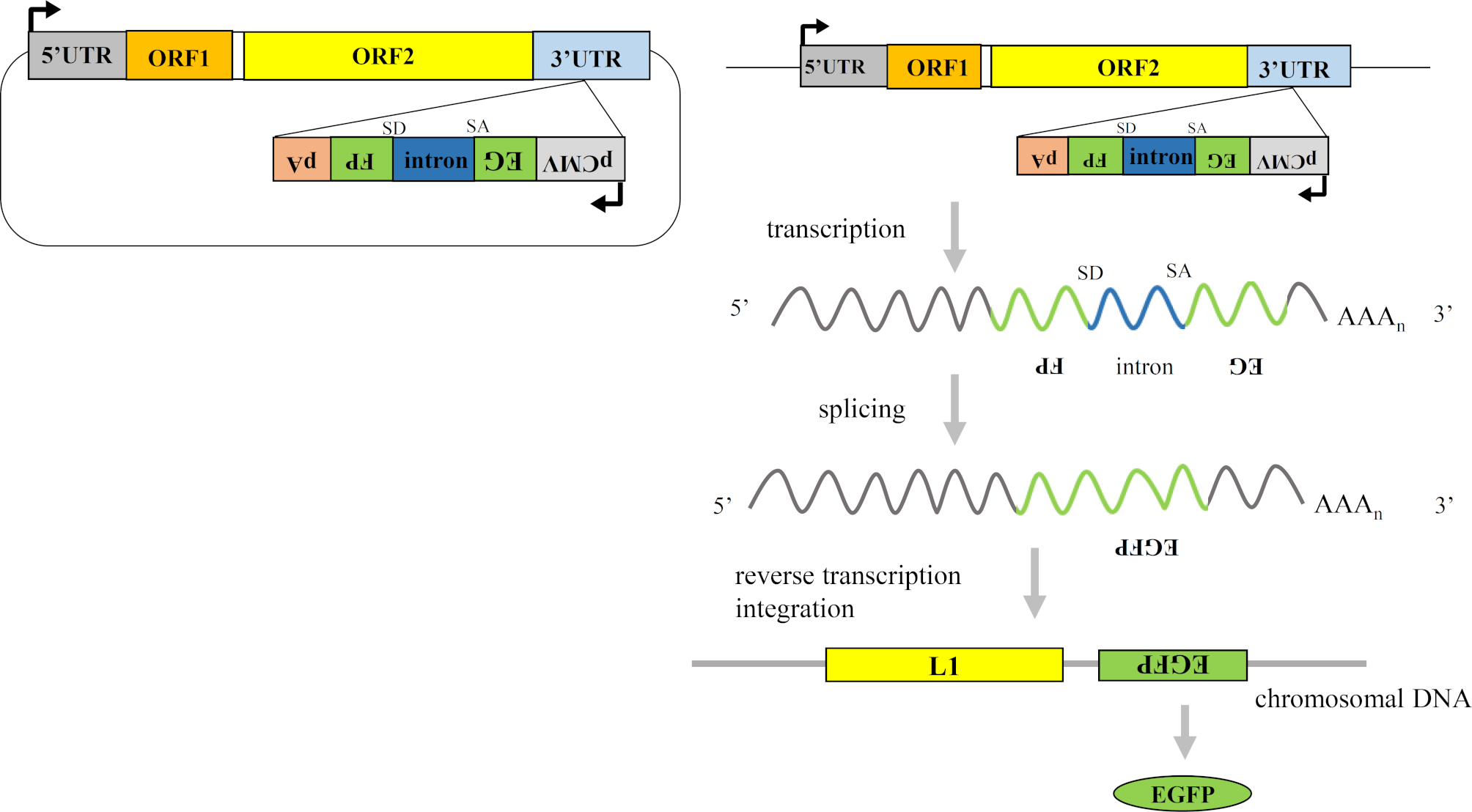

Supplement: Supplementary file 5 — Figure S5. Schematic diagram of the pLRE3-mEGFP1 construct (left) and rationale of the L1-retrotransposition assay (right). The EGFP retrotransposition reporter cassette is cloned into the 3′UTR of L1 in the antisense orientation. The cassette consists of the CMV promoter (pCMV), the TK poly(A) signal (pA) and the EGFP gene interrupted by a sense orientation intron (intron) with the splice donor (SD) and splice acceptor (SA). (TIF 335 kb) [file 13148_2019_661_MOESM5_ESM.tif]
